# Higher TNF-α, IGF-1, and Leptin Levels are Found in Tasters than Non-Tasters

**DOI:** 10.3389/fendo.2014.00125

**Published:** 2014-07-29

**Authors:** Rui Wang, Nika M. A. van Keeken, Sana Siddiqui, Lea M. Dijksman, Stuart Maudsley, Diana Derval, P. Sytze van Dam, Bronwen Martin

**Affiliations:** ^1^Metabolism Unit, National Institute on Aging, National Institutes of Health, Baltimore, MD, USA; ^2^Department of Internal Medicine, Onze Lieve Vrouwe Gasthuis, Amsterdam, Netherlands; ^3^Receptor Pharmacology Unit, National Institute on Aging, National Institutes of Health, Baltimore, MD, USA; ^4^Teaching Hospital, Onze Lieve Vrouwe Gasthuis, Amsterdam, Netherlands; ^5^VIB Department of Molecular Genetics, University of Antwerp, Antwerp, Belgium; ^6^Better Immune System Foundation, Amsterdam, Netherlands

**Keywords:** taste, TNF-α, leptin, IGF-1, BMI

## Abstract

Taste perception is controlled by taste cells that are present in the tongue that produce and secrete various metabolic hormones. Recent studies have demonstrated that taste receptors in tongue, gut, and pancreas are associated with local hormone secretion. The aim of this study was to determine whether there is a link between taste sensitivity and levels of circulating metabolic hormones in humans and whether taste sensitivity is potentially related to peripheral metabolic regulation. Thirty-one subjects were recruited and separated into tasters and non-tasters based on their phenol thiocarbamide (PTC) bitter taste test results. Fasting plasma and saliva were collected and levels of hormones and cytokines were assayed. We observed significant differences in both hormone levels and hormone-body mass index (BMI) correlation between tasters and non-tasters. Tasters had higher plasma levels of leptin (*p* = 0.05), tumor necrosis factor-α (TNF-α) (*p* = 0.04), and insulin-like growth factor 1 (IGF-1) (*p* = 0.03). There was also a trend toward increased IGF-1 levels in the saliva of tasters (*p* = 0.06). We found a positive correlation between plasma levels of glucose and BMI (*R* = 0.4999, *p* = 0.04) exclusively in non-tasters. In contrast, plasma C-peptide levels were found to be positively correlated to BMI (*R* = 0.5563, *p* = 0.03) in tasters. Saliva TNF-α levels were negatively correlated with BMI in tasters (*R* = −0.5908, *p* = 0.03). Our findings demonstrate that there are differences in circulating levels of leptin, TNF-α, and IGF-1 between tasters and non-tasters. These findings indicate that in addition to the regulation of food consumption, taste perception also appears to be tightly linked to circulating metabolic hormone levels. People with different taste sensitivity may respond differently to the nutrient stimulation. Further work investigating the link between taste perception and peripheral metabolic control could potentially lead to the development of novel therapies for obesity or Type 2 diabetes.

## Introduction

The sense of taste is critical for humans to detect nutritionally relevant and harmful compounds in food (1). Human taste sensations are mainly categorized as: sweet, sour, salty, bitter, and umami. In addition to the tongue, taste receptors are also present in multiple peripheral organs including gut, pancreas and also the brain (2). Studies have shown that in the gut, the secretion of satiation peptides (GLP-1 and PYY) is regulated by the type 1 taste receptor T1R2/T1R3 heterodimers (3, 4), whereas in the pancreatic β-cells, the T1R1/T1R3 heterodimers regulate autophagy and insulin secretion (2, 5). In taste cells, hormones such as PYY, glucagon, and GLP-1 have also been reported to be present specifically in T1R3-positive cell types, indicating a close relationship between these hormones and taste sensitivity (6–9). These metabolic and appetite hormones regulate taste responsiveness in a paracrine manner and thus can regulate food intake behavior. Therefore, taste perception and taste receptor expression are associated with metabolic and appetite hormones and may regulate peripheral metabolic control (10, 11).

TNF-α is highly expressed in type 2 taste cells and is co-localized with the T1R3 receptor subunit (12, 13). TNF-α mRNA has been shown to be fivefold higher in the taste epithelium than in the non-taste epithelium in mouse. Such high abundance suggests that it regulates local immune response and taste sensation (12). On the other hand, TNF-α that is expressed in adipose tissue also plays a role in regulating insulin sensitivity in obesity-induced insulin resistance and type 2 diabetes (14). However, thus far, little has been reported on TNF-α levels in people who have different taste sensitivity.

Growth hormones in both brain and peripheral organs regulate food intake, nutrient absorption, and energy metabolism (15–17). Many of these hormones and their receptors are found in the tongue and saliva, including insulin-like growth factor 1 (IGF-1) and brain-derived neurotrophic factor (BDNF) (18, 19). The presence of BDNF and its receptor TrkB in the tongue are necessary for taste bud formation during development (20). IGF-1 is also present in taste cells, together with insulin receptors, IGF-2 and IGFBP (insulin-like growth factor binding protein). Their presence might also be associated with cell growth in papilla (19).

Presence of the taste receptors in the gastrointestinal system and their relationship with gut hormone secretion suggest that there is a close connection between taste perception and food digestion and absorption (21, 22). Similarly to the tongue, the taste receptors T1R2 and T1R3 are co-localized with multiple gut hormones in the intestine (3). Lack of T1R3 or its downstream G protein α-gustducin results in decreased glucose stimulated GLP-1 secretion (23). The mRNA levels of the T1R2 receptor decrease during hyperglycemia in healthy human subjects but increase under the same condition in type 2 diabetic patients, potentially resulting in increased glucose absorption in these patients (24). In addition, T1R2, T1R3, and their downstream signaling molecules are also expressed in pancreatic β-cells and MIN6 insulinoma cells. These receptors regulate insulin secretion from the MIN6 cells by activating calcium and cAMP signaling pathway (5). However, whether there is a difference in hormone levels amongst people who have differences in taste sensitivity is not clear. The aim of this study was to determine whether there is a link between taste sensitivity and levels of circulating hormones in human subjects. We found differences in levels of plasma leptin, TNF-α, and IGF-1 between tasters and non-tasters. Our study revealed the associations between taste perception and hormone levels in both plasma and saliva. These findings provide a foundation for future studies to further describe the metabolic differences between the two taste groups.

## Materials and Methods

### Subjects

This study was approved by the institutional review boards of the National Institute on Aging (Baltimore, MD, USA) and the Onze Lieve Vrouwe Gasthuis (Amsterdam, The Netherlands), and all participants gave written informed consent prior to each assessment. Thirty-one non-diabetic healthy male subjects were recruited and their medical history and background information were collected. To avoid any possible influences that could lead to hormone level and taste perception changes, we introduced a series of inclusion and exclusion criteria when recruiting subjects (Table 1). Subjects were given a standardized PTC test, during which a piece of paper strip impregnated with 0.3 mg/pc PTC was provided. The subjects were asked to put the strip on their tongue allowing it to moisten. Subjects who reported a bitter taste, were classified as “tasters” and subjects who didn’t perceive the bitterness were classified as “non-tasters” (25). Subjects’ height, weight, and waist circumference were measured and body mass index (BMI) was calculated as weight (kg)/[height (m)]^2^.

**Table 1:** **Subject recruiting criteria**.

Inclusion criteria	Exclusion criteria
Male	Age below 30 years or over 55 years
Caucasian	BMI lower than 18.5 kg/m^2^ or higher than 31 kg/m^2^
Age between 30–55	Psychiatric morbidity (eating disorder, depression, alcoholism)
	Co-morbidity (cardiovascular disease, thyroid disease, diabetes, obesity)
	Previous history of cancer
	Use of regular anti-inflammatory medication
	Consumption of diets or supplements high in phytoestrogens
	Smoking

*BMI, body mass index, calculated as weight (kg)/[height (m)]^2^*.

### Measurement of circulatory and saliva hormones

Plasma and saliva samples were collected in the morning after an overnight fast. For saliva collection, we used Sarstedt salivette (Sarstedt, Germany) according to the manufacturer’s instructions. Briefly, after a subject chewed on a swab for 45 s the swab was placed into a tube and the saliva sample was obtained by centrifuging at 2000 rpm (4°C) for 10 min. Blood was collected in standard EDTA-containing sampling tubes, then centrifuged at 3000 rpm, 4°C for 30 mins. Samples were then stored at −80°C until further processing. Levels of amylin (total), C-peptide, glucose-dependent insulinotropic peptide (GIP), insulin, TNF-α, leptin, PP, and PYY were measured using the MILLIPLEX MAP human metabolic hormone magnetic bead panel (EMD Millipore Corporation, Billerica, MA, USA): intra-assay variation was 2–8% (26). Adiponectin levels were measured using a Millipore ELISA kit, the intra-assay variation was 1.0–7.4%. BDNF and IL-6 were assayed using R&D system ELISA kits (Minneapolis, MN, USA): intra-assay variations were 3.8–6.2 and 1.6–4.2%. IGF-1 was measured by ELISA assay (Alpco Diagnostics, Salem, NH, USA): intra-assay variation was 5.08–6.65%. Blood glucose levels were measured using a glucose assay kit (Cayman Chemical Company, Ann Arbor, MI, USA): intra-assay variation was 4.6–8.1%. All hormone and glucose levels were derived by interpolation from reference curves generated in the same assays with reference standards of known concentrations. HOMA-IR was calculated using glucose (mg/dL) × insulin (mU/L)/405(16, 26).

### Measurement of plasma free fatty acids and triacylglycerol

Free fatty acid and triacylglycerol levels were measured using enzymatic assay kits according to the manufacturer’s instructions (Wako Pure Chemical Industries, Ltd., Japan). Free fatty acid and triacylglycerol levels were derived by interpolation from reference curves generated in the same assays with reference standards of known concentrations (26).

### Statistical analysis

For hormone, lipid, and glucose level measurements, data were log-transformed to achieve normal distributions for all analyses, and then unpaired *t*-tests were performed using GraphPad Prism V5, in which *p* < 0.05 was considered statistically significant. Spearman correlation coefficients were calculated to test the correlation between hormone levels and BMI. A value of *p* < 0.05 was considered statistically significant with respect to the linear correlations’ deviation from a zero slope.

## Results

### Plasma hormone, cytokine levels, and lipid profile in tasters and non-tasters

**Figure 1:**
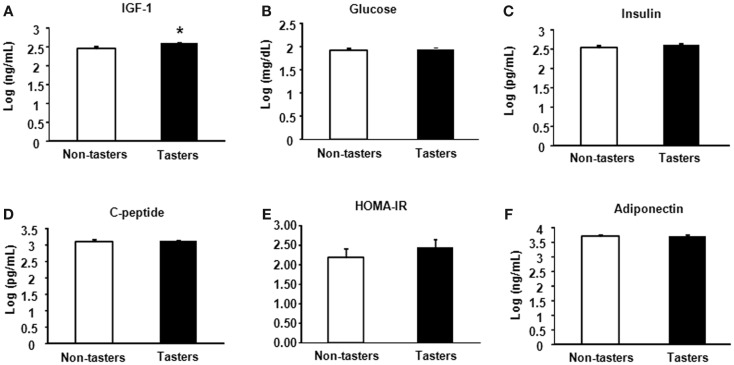
**Plasma metabolic hormones of non-tasters and tasters**. Plasma samples from non-tasters and tasters were assayed to measure the levels of IGF-1 **(A)**, glucose **(B)**, insulin **(C)**, C-peptide **(D)**, HOMA-IR **(E)**, and Adiponectin **(F)**. Results were log-transformed and subjected to a non-paired two-tailed *t*-test. A value of *p* < 0.05 was considered statistically significant.

**Figure 2:**
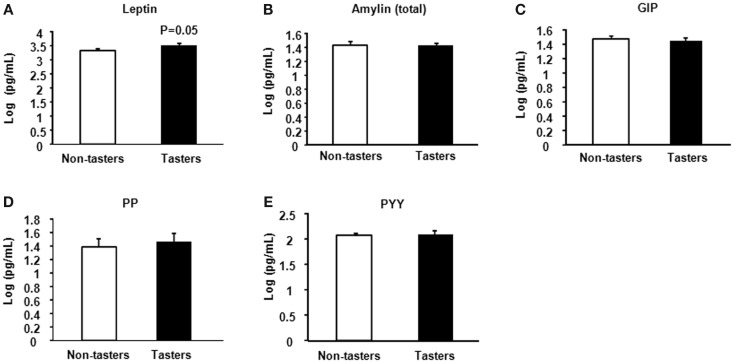
**Plasma appetite hormones**. Appetite hormones including leptin **(A)**, amylin (total) **(B)**, GIP **(C)**, PP **(D)**, and PYY **(E)** were measured. Results were log-transformed and subjected to a non-paired two-tailed *t*-test. A value of *p* < 0.05 was considered statistically significant.

**Figure 3:**
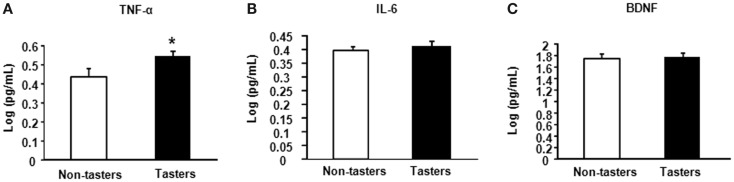
**Plasma levels of inflammatory and growth factors**. The inflammatory factors TNF-α **(A)**, IL-6 **(B)**, and brain-derived neurotrophic factor BDNF **(C)** were measured. Results were log-transformed and subjected to a non-paired two-tailed *t*-test. A value of *p* < 0.05 was considered statistically significant.

After dividing our 31 male subjects into tasters and non-tasters, no significant differences in age, BMI or waist circumference between the two groups were observed (Table 2). Plasma levels of metabolic hormones are shown in Figure 1, we found that the plasma IGF-1 levels of tasters were significantly higher than those of non-tasters (Figure 1A). Glucose, insulin, C-peptide, HOMA-IR, and adiponectin levels were not different between tasters and non-tasters (Figures 1B–F). We observed higher plasma leptin levels in tasters than in non-tasters (Figure 2A, *p* = 0.05). No differences in the levels of total amylin, GIP, PP, or PYY were found (Figures 2B–E). The plasma TNF-α levels in tasters were higher than in non-tasters (Figure 3A). For IL-6 and BDNF, no differences were found between tasters and non-tasters (Figures 3B,C). In addition, free fatty acid and triacylglycerol levels were measured. No differences were found considering these two lipid levels (Figure S1 in Supplementary Material).

**Table 2:** **Subject characteristics**.

Factors	Tasters	Non-tasters
Age (years)	45.8 ± 1.86	40.8 ± 1.93
BMI (kg/m^2^)	24.7 ± 0.53	25.3 ± 0.56
Waist circumference (cm)	89.6 ± 1.51	89.2 ± 1.71

*Data are shown as mean ± SEM*.

### Saliva hormone and cytokine levels in tasters and non-tasters

**Figure 4:**
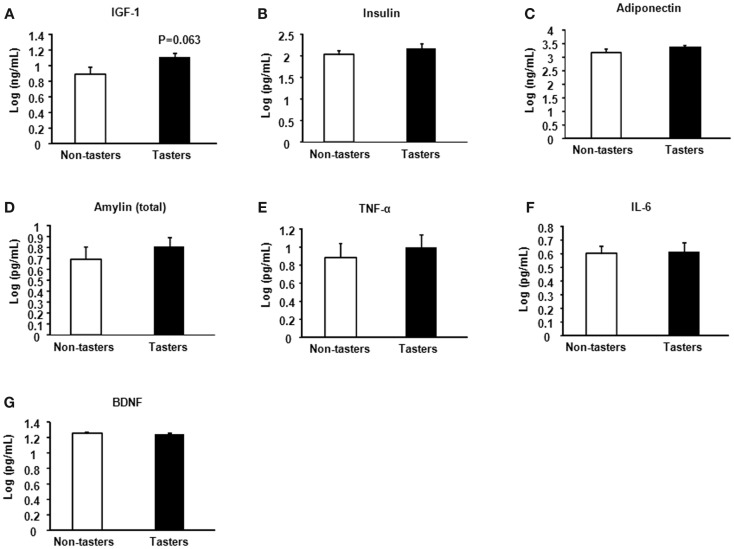
**Saliva hormones and cytokines of non-tasters and tasters**. Saliva samples from non-tasters and tasters were assayed to measure the levels of IGF-1 **(A)**, insulin **(B)**, adiponectin **(C)**, amylin **(D)**, TNF-α **(E)**, IL-6 **(F)**, and BDNF **(G)**. Results were log-transformed and subjected to a two-tailed *t*-test. A value of *p* < 0.05 was considered statistically significant.

A trend toward increased IGF-1 was found in tasters compared with non-tasters (Figure 4A), which was similar to what we observed in the plasma samples. No differences were found in the levels of insulin, adiponectin, amylin, TNF-α, IL-6, or BDNF (Figures 4B–G).

### Correlations between plasma and saliva hormone levels and BMI

**Figure 5:**
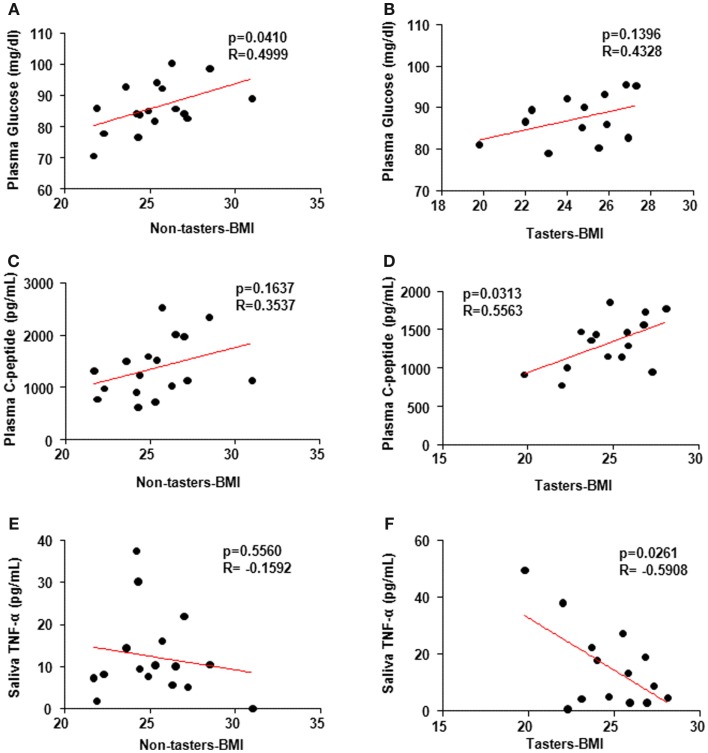
**Spearman’s correlation coefficient analysis correlating plasma and saliva factors and body mass index (BMI)**. Plasma glucose levels **(A,B)** were correlated with BMI in non-tasters but not tasters. C-peptide was correlated with BMI in tasters but not non-tasters **(C,D)**. Saliva TNF-α was reversely correlated with BMI in tasters but not non-tasters **(E,F)**. A value of *p* < 0.05 was considered statistically significant.

It is not clear whether BMI association with the hormones is taste-sensitivity dependent. Spearman’s correlation coefficient using individual hormone levels versus the BMI revealed a positive correlation between glucose levels and BMI in non-tasters (Figure 5A, *p* = 0.04, R-0.4999) but not in tasters (Figure 5B, *p* = 0.14, *R* = 0.4328). Additionally, a positive correlation between plasma C-peptide and BMI was found in tasters (Figure 5D, *p* = 0.03, *R* = 0.5563) but not in non-tasters (Figure 5C, *p* = 0.16, *R* = 0.3537). We found a significant negative correlation between saliva TNF-α levels and BMI (Figure 5F, *p* = 0.02, *R* = −0.5908) in tasters, whereas in non-tasters no correlation was observed (Figure 5E, *p* = 0.56, *R* = −0.1592).

## Discussion

Our study of hormones as well as cytokines in plasma and saliva samples of tasters and non-tasters showed differences in levels of plasma IGF-1, leptin, and TNF-α. Specifically, higher levels of all three factors were observed in tasters than in non-tasters. A positive correlation between plasma glucose levels and BMI was observed in non-tasters, whereas a positive correlation between C-peptide and BMI was observed in tasters. Interestingly, a negative correlation between saliva TNF-α and BMI was observed in tasters.

A well-established method classifying tasters and non-tasters by assessing the sensitivity to the bitter chemical phenylthiocarbamide (PTC) is widely used in both research and food industrial fields (27–29). Due to its high sensitivity, in the current study, we chose PTC taste to classify our subjects into tasters and non-tasters. Studies show that the bitter taste threshold in young adults is correlated with the thresholds of sucrose, sodium chloride, quinine-HCl, and monosodium glutamate perceptions (29, 30). Despite the taste receptor differences, bitter and sweet taste transductions share the same downstream G protein signaling pathway (31).

Recent studies have reported the presence of hormones regulating food intake and energy metabolism in oral tissues (6, 32–34). In our study, we found higher circulating levels of leptin in the tasters. The function of leptin has been well demonstrated as a controller of food intake, energy expenditure, and body weight (35, 36). In the hypothalamus, injection of leptin inhibits food intake by inhibiting the activity of AMP-activated kinase (AMPK) and by activating acetyl-CoA carboxylase (ACC) (37). Leptin receptors are also expressed in mouse circumvallate papilla (38). Non-functional leptin receptor mutant mice (*db*/*db*) display a higher preference for sweet taste than do wild-type mice and administration of leptin suppresses this sweet taste sensitivity (38). Although in this pilot study, we did not measure leptin receptor expression in the taste buds of the subjects, the increased leptin levels suggest that differences in the control of energy homeostasis could exist between tasters and non-tasters.

The pro-inflammatory cytokine TNF-α is mainly secreted from monocytes, macrophages, and adipocytes (39, 40). Adipose tissue is reported to act as an endocrine organ, sensing and regulating energy status, and activating signals in other organs to maintain energy balance (41). TNF-α plays a critical role by regulating fatty acid uptake, lipogenesis, and lipolysis in adipocytes (41). We found higher levels of plasma TNF-α in tasters than in non-tasters, but these higher levels were still within a relatively normal range (42). Furthermore, saliva TNF-α levels were negatively correlated with BMI in tasters but not in non-tasters. TNF-α expression in the type 2 taste cells has been reported to contribute to local immune surveillance given the observation of rapid increase of TNF-α levels in these taste cells upon inflammation challenge (12). Because immune cells are rarely found in the taste buds (43), the level of TNF-α in the saliva to some extent reflects the amount and function of type 2 taste cells. Therefore, our observation of the negative correlation between TNF-α and BMI suggests that in tasters lower BMI could be related to a better taste cell function and taste perception. Whereas in non-tasters, there is no such relation exists.

No report to date has conclusively shown whether taste sensitivity in humans is associated with body weight and insulin sensitivity. In this study, we excluded subjects with metabolic disorders, thus, the levels of glucose, insulin, and C-peptide were not different between tasters and non-tasters. However, we observed a positive correlation between plasma glucose and BMI in non-tasters but not tasters, suggesting that plasma glucose levels are more affected by body weight in non-tasters than in tasters. Additionally, the C-peptide levels in tasters were correlated with BMI. C-peptide is produced from proinsulin in pancreatic β-cells and links peptides A and B of insulin (44). C-peptide is released simultaneously with insulin from β-cells and yet has a longer half-life than insulin. Thus, the plasma C-peptide level is used as a more reliable indicator of pancreatic β-cell secretion function (45). We found the C-peptide levels of tasters to be correlated with BMI, raising the interesting possibility that β-cell secretory function could be more sensitive to body weight changes in these subjects. Although, both tasters and non-tasters in this study exhibited normal plasma glucose and insulin levels. The correlation between glucose and BMI in non-tasters and the correlation between C-peptide and BMI in tasters imply that when considering the body weight effect on the glucose stimulated insulin secretion, tasters may have higher sensitivity to blood glucose change, and stimulate insulin secretion better to cope with the increased need.

Additionally, we observed increased plasma IGF-1 levels in the tasters and this same trend was also found in saliva. Studies show that the association between IGF-1 levels and diabetes risk form a U-shaped curve, indicating that people with either low or high levels of IGF-1 possess a higher risk of developing type 2 diabetes (46). The role of IGF-1 in the metabolic function of tasters and non-tasters remains unclear and further studies are needed to investigate this. In summary, our study of the hormones in people with different taste sensitivities suggests a close relationship between taste perception and hormone secretion. People with different taste sensitivity may have differences in their response to the nutrient stimulation and in their metabolic hormone secretion. In general, tasters exhibited higher levels of leptin, TNF-α, and IGF-1 than non-tasters. Our study also indicates that the taste perception, in addition to its role of directly control food consumption, may also affect food absorption, digestion, and energy metabolism by affecting hormone levels in human. Due to the limitation of sample size and exclusion of subjects with metabolic disorders in the current study, we did not observe any differences in the levels of plasma glucose, insulin, or HOMA-IR. Recent studies of taste receptors controlling the hormone release in both the gut and pancreas have set a focus on the importance of these receptors in the metabolic function of the body. However, more work still needs to be done to uncover the potential role of taste perception in human metabolic control and we hope that our study will encourage further work concerning this topic.

## Conflict of Interest Statement

The authors declare that the research was conducted in the absence of any commercial or financial relationships that could be construed as a potential conflict of interest.

## Supplementary Material

The Supplementary Material for this article can be found online at http://www.frontiersin.org/Journal/10.3389/fendo.2014.00125/abstract
