# Supplementary material for: Higher TNF-α, IGF-1, and Leptin Levels are Found in Tasters than Non-Tasters
Source: Front Endocrinol (Lausanne). 2014 Jul 29;5:125. doi: 10.3389/fendo.2014.00125 (PMC4114300; doi:10.3389/fendo.2014.00125)
Supplement: Supplementary file 1 [file Presentation_1.PDF]

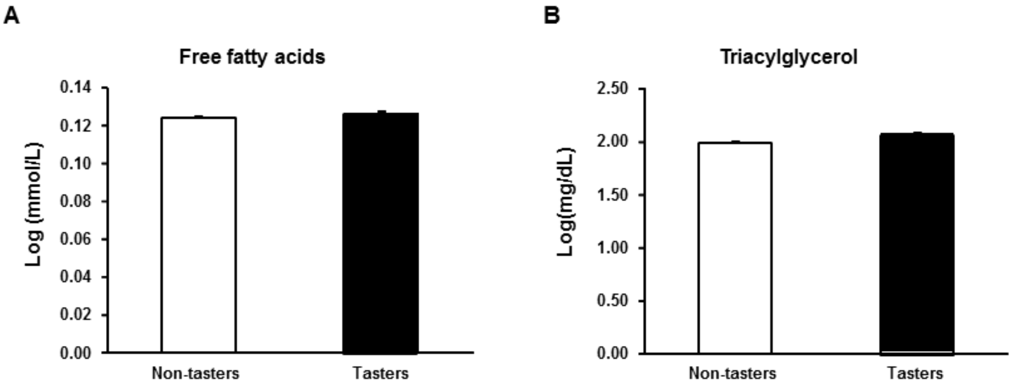

Figure S1

Figure S1. Plasma lipid profile.

Plasma free fatty acids and triacylglycerol levels were measured. Results were log-transformed and subjected to a two-tailed t-test. A value of $p<0.05$ was considered statistically significant. There was no difference between tasters and non-tasters in either free faty acid levels (A) or triacylglycerol levels (B).
